# Parents’ Attitudes towards Vaccinations Regarding the Ukrainian Migration to Poland in 2022

**DOI:** 10.3390/vaccines11081306

**Published:** 2023-07-31

**Authors:** Martyna Cholewik, Maciej Stępień, Carlo Bieńkowski, Maria Pokorska-Śpiewak

**Affiliations:** 1 Student Scientific Circle at the Department of Children’s Infectious Diseases, Medical University of Warsaw, Wolska 37, 01-201 Warsaw, Poland; s077260@student.wum.edu.pl (M.C.); s073965@student.wum.edu.pl (M.S.); 2 Doctoral School, Medical University of Warsaw, Żwirki i Wigury 61, 02-091 Warsaw, Poland; 3 Department of Children’s Infectious Diseases, Medical University of Warsaw, Wolska 37, 02-091 Warsaw, Poland; maria.pokorska-spiewak@wum.edu.pl; 4 Hospital of Infectious Diseases, 01-201 Warsaw, Poland

**Keywords:** Ukraine, international migration, vaccination coverage, parents, vaccination hesitancy

## Abstract

*Background:* Russia’s aggression against Ukraine in early 2022 resulted in a large migration of refugees to many countries, including Poland. Vaccination coverage for some infectious diseases in Ukraine is lower than in Poland; consequently, the incidence of infectious diseases—including measles, poliomyelitis, tuberculosis, and COVID-19—is higher. We aimed to investigate whether the migration of Ukrainians had influenced decisions of Polish parents on having their children vaccinated and to examine their attitudes towards vaccinations. *Material and methods:* A cross-sectional online survey study was designed. Data on parents’ demographics, attitudes toward vaccination, and knowledge of the current epidemiological situation in Poland were collected. Parents participating in the study were divided into two subgroups for further analysis according to their decisions to have their children vaccinated. *Results*: A total of 568 questionnaires were collected, of which 21 did not meet the inclusion criteria for the analysis (respondents were not parents). The Ukrainian immigrants’ influx affected 54 (9.87%) participants in their decision of having their children vaccinated. Respondents in this group were more likely to have a positive attitude toward recommended vaccinations (*p* = 0.0428); in addition, they more often had their children vaccinated with recommended vaccinations (*p* = 0.0063), believed the vaccination coverage with mandatory vaccinations was higher in Poland than in Ukraine (*p* = 0.0014), and believed the incidence of diseases covered by mandatory (*p* = 0.0472) and recommended (*p* = 0.0097) vaccinations was higher in Ukraine. In addition, parents who declared that the migration had affected their decision regarding their children’s vaccinations had more often been vaccinated due to the influx of Ukrainian immigrants (*p* < 0.00001) and were more likely to be aware of how migration had impacted the current epidemiological situation in Poland (*p* = 0.0021). Moreover, the survey more often made these participants think about getting additional vaccinations for themselves (*p* < 0.0001) and their children (*p* < 0.0001). *Conclusions*: The Ukrainian immigrants’ influx affected nearly one tenth of surveyed parents in their decision of having their children vaccinated. This group was more aware of the differences between infectious diseases’ epidemiology in Poland and Ukraine. In addition, they also had a more positive attitude toward vaccinations.

## 1. Introduction

Russia’s aggression against Ukraine in February 2022 resulted in a large migration of refugees to many countries. As of January 2023, an estimated 9.5 million people have crossed the Polish border since the war began [1]. Among them, many have settled in Poland. The level of vaccination and incidence rate of certain infectious diseases in Ukraine is described by the World Health Organization (WHO) as alarming. In particular, these include measles, polio, tuberculosis, and COVID-19 [2]. Since the beginning of the COVID-19 pandemic, there has been a lot of misinformation in Ukraine, leading to a low vaccination rate against the SARS-CoV-2 virus [3]. On the day the war broke out, only 34% of the population was vaccinated [4]. Measles outbreaks were also reported in Ukraine in the second decade of the 21st century due to a sharp decline in public confidence in vaccinations after the crisis and war in 2010. In 2018, the number of cases exceeded 54,000 [5]. In 2002, the European region was declared a polio-free area. However, in 2015, two unrelated cases of children being paralyzed by the wild poliovirus were reported in Ukraine [6]. In addition, tuberculosis is becoming a growing problem, making Ukraine the fifth country in the world with the highest number of extensively drug-resistant confirmed cases [7]. Factors that compound the risk of an epidemic include poor sanitation and overcrowding in refugee asylums [8]. In March 2022, the WHO identified the most needed vaccinations for refugees, which included vaccinations against COVID-19; measles, mumps, and rubella (MMR); diphtheria, tetanus, and pertussis (DTP); poliomyelitis; and *Hemophilus Influenzae type B* (Hib). Hepatitis B, meningococcus, pneumococcus, chickenpox, influenza, and tuberculosis should also be considered depending on the season, epidemiological situation, and living conditions [2]. The above might pose a major public health challenge in Poland [9]. This is the reason why vaccination should be especially promoted among both Polish citizens and migrants from Ukraine. Mandatory and recommended vaccinations in Poland are regulated by the immunization program and are shown in Table 1. We aimed to investigate whether the migration of Ukrainians had influenced parents’ decisions on having their children vaccinated and to examine their attitudes towards vaccinations. We focused on comparing the population that declared the impact of Ukrainian migration on their children’s vaccination decisions with the group that did not.

## 2. Materials and Methods

### 2.1. Study Design and Participants

A cross-sectional survey study was designed. The questionnaire included 25 single-choice and 4 multiple-choice questions on demographics (gender, age, place of residence, and education), whether respondents were parents of a child/children under 18, their attitudes toward vaccination, and their knowledge of the current epidemiological situation in Poland. The division into mandatory and recommended vaccinations was carried out on the basis of the law regulating the program of immunization in Poland in 2023.

Data were collected through an online survey, which was created using the “Google Forms” application between 15 November 2022 and 4 January 2023 and distributed via social media (Facebook and Instagram) on forums and social media groups/accounts related to parenting issues. Each participant was asked to complete the survey only once. There was no time limit to access the form. After completing the survey, participants received feedback on the epidemiological situation in Ukraine and Poland for education purposes.

After collecting responses, the study group was divided into two subgroups for further analysis. Stratification was performed according to the declared impact of Ukrainian migration on participants’ decisions as to whether they would vaccinate their children or not.

### 2.2. Inclusion Criteria

Inclusion criteria were completing the form and being a parent of at least one child under the age of 18.

By completing the questionnaire, each respondent agreed to anonymously participate in the study and gave consent for publication of the results of this study. 

### 2.3. Exclusion Criteria

The exclusion criterion considered the indication that one is not a parent of a child/children under 18 years of age. Twenty-one surveys were rejected because respondents answered that they were not parents.

### 2.4. Statistical Analysis

The Mann–Whitney U test was performed to compare continuous variables and the chi^2^ or Fisher exact test were used to evaluate categorical variables. A *p*-value of <0.05 was considered significant. Statistical analysis was performed using Quick Statistics Calculators (available at https://www.socscistatistics.com, accessed on 20 January 2023).

## 3. Results

In total, 568 questionnaires were collected, of which 21 were excluded due to not meeting the inclusion criteria. The median age of the study participants was 34 (interquartile range (IQR): 31–39 years). The majority were females (503/547 (91.96%). Cities with over 500,000 inhabitants were the place of residence for 179/547 (32.72%) respondents, and a college degree was obtained by 472/547 (82.29%).

A positive attitude toward vaccination was declared by 497/547 (90.86%) study participants. According to the surveyed population, the Ukrainian immigrants’ influx had affected 54/547 (9.87%) people in their decision of having their children vaccinated. No influence on this decision was declared by 493/547 (90.12%) participants.

Respondents whose vaccination decisions were influenced by the migration of Ukrainians were more likely to have a positive attitude toward the use of recommended vaccinations (49/54, 90.74% vs. 433/497, 87.23%; *p* = 0.0428), have their children vaccinated with recommended vaccinations (47/54, 87.04% vs. 401/493, 81.34%; *p* = 0.0063), were more likely to believe the vaccination rate with mandatory vaccinations was higher in Poland than in Ukraine (49/54, 90.74% vs. 384/493, 77.89%; *p* = 0. 0014), believed that the incidence rate of diseases covered by mandatory vaccinations was higher in Ukraine than in Poland (48/54, 88.89% vs. 364/493, 73.83%; *p* = 0.0472), believed the incidence rate of diseases covered by recommended vaccinations was higher in Ukraine than in Poland (49/54, 90.74% vs. 353/493, 71.60%; *p* = 0.0097), were more likely to have been vaccinated due to the influx of Ukrainian immigrants (17/54, 31.48% vs. 9/493, 1.83%; *p* < 0.00001), and were more likely to be aware of how the influx of migrants from Ukraine had affected the epidemiological situation in Poland (40/54, 74.07% vs. 271/493, 54.97%; *p* = 0.0021). More often than not, the survey made participants whose vaccination decisions were influenced by the migration of Ukrainians think about getting additional vaccinations (41/54, 75.93% vs. 164/493, 33.27%; *p* < 0.0001) and having their children vaccinated with additional vaccines (44/54, 81/48% vs. 162/493, 32.86%; *p* < 0.0001) (See Table 2 and Table 3).

There was no significant difference in vaccines chosen by parents between the two groups. Participants most often chose for themselves to be vaccinated against pertussis (19, 3.47%), SARS-CoV-2 (18, 3.29%), and influenza (16, 2.93%), while they intended to be vaccinated against influenza (16, 2.93%), SARS-CoV-2 (10, 1.83%), meningococcal disease (8,1.46%), and pertussis (8, 1.46%). In an analogous question about the participants’ children, most children had already been vaccinated against meningococcal disease (18, 3.29%), influenza (18, 3.29%), and varicella (16, 2.93%). Likewise, their parents most often intended to have them vaccinated against varicella (14, 2.56%), meningococcal disease (14, 2.56%), and influenza (13.2.38%).

## 4. Discussion

The problem of evading mandatory vaccinations is growing worldwide, including Poland. It poses a huge threat to the public health in this country. In 2015, in Poland, infectious diseases caused about 1900 deaths, of which 300 deaths were due to hepatitis B and more than 500 due to pulmonary tuberculosis. Both diseases may be effectively prevented with mandatory vaccinations implemented within the first year of life [11]. Between 2015 and 2019, the percentage of children in Poland who were not vaccinated as a result of their parents’ conscious decision tripled. The rate of refusal to vaccinate children and adolescents per 1000 people was 2.3 in 2015, 6.6 in 2019, and 8.3 in 2021 [12,13]. A particularly visible effect of such a trend was the sharp increase in measles cases in 2018 and 2019 [14,15]. According to Szalonek A., fear of complications after vaccination is the most common reason for parents to avoid vaccinating their children [16]. In addition, strong anti-vaccine beliefs are related to respondents’ deep religiosity and living in a city with a small or medium population [17]. In reference to a study conducted by Kraśnicka J. et al., Poles’ attitudes toward mandatory vaccinations vary widely by age. There is a big difference between 41–50-year-olds and 18–30-year-olds, with a much smaller proportion of younger parents supporting mandatory immunization (OR = 0.352) [18]. In our survey, there were no significant differences in vaccinating children according to the guidelines among respondents whose vaccination decisions were influenced by the influx of Ukrainian immigrants and those who were not (53/54, 98.15% vs. 484/497, 98.17%; *p* = 0.9891).

The following Polish studies examined parents’ feelings about recommended vaccines: according to Janosz J. et al., a positive attitude towards the recommended vaccinations was recorded in 60% of the parents who had children under 3 years of age [19]; according to Lipska E. et al., 44% of surveyed parents had their children vaccinated with at least one recommended vaccine [20]; according to Pisaniak P. et al., 66.41% of the parents would reach for the recommended vaccinations for their children if they were free of charge. Nevertheless, in that study, respondents who refused to have their children vaccinated would not like to receive the vaccines, even if they were free [21]. A Polish large survey study revealed that the majority of women wanted to be vaccinated and have their children vaccinated, and considered vaccines to be safe and effective against infectious diseases [22]. According to Świątoniowska N.A. et al., 60.8% of mothers declared that it is worth vaccinating their children with recommended vaccinations [23]. In our survey, 482/547 (88.12%) participants specified that they were in favor of the implementation of recommended vaccines. In addition, respondents whose decisions regarding vaccinations were influenced by the migration of Ukrainians more frequently had their children vaccinated with recommended vaccinations (47/54, 87.04% vs. 401/493, 81.34%; *p* = 0.0063).

Male parents, parents who had a university education, and parents who had not been exposed to adverse reactions after vaccination in the past were more likely to report that their children have received the recommended vaccinations [18]. Moreover, parents who vaccinated their children with recommended vaccines showed significantly higher levels of health practices (daily habits regarding sleep and rest) compared with those who did not have their children vaccinated with these vaccines [24]. A study conducted by Ganczak M. et al. on factors influencing parents’ willingness to vaccinate their children against Human Papilloma Virus (HPV), which is one of the recommended vaccinations, showed that mothers were more likely to have their children vaccinated than fathers (90.8% vs. 82.8%, *p* = 0.03). The study also showed that working parents were more likely to report willingness to have their children vaccinated compared with unemployed parents (87.0% vs. 79.0%, *p* = 0.05). In contrast, no differences were found in terms of age, place of residence, education level, marital status, number of children in the family, or religiosity [25]. Dąbek J. et al. showed an association between the use of recommended vaccination and age (*p* = 0.002) or education level (*p* = 0.004). Among respondents using recommended vaccinations, those aged 18–30 years and those with tertiary education had the highest proportion [26]. In our survey, respondents whose vaccination decisions were influenced by the migration of Ukrainians were more likely to have a positive attitude toward the use of recommended vaccinations (49/54, 90.74% vs. 433/497, 87.23%; *p* = 0. 0428).

The influx of many refugees from Ukraine may pose a potential epidemiological threat. It is associated with infectious diseases including, e.g., COVID-19, measles, and poliomyelitis [27]. Rzymski P. et al. showed that by February 24, 2022, only 35% of the Ukrainian population had been vaccinated with the complete initial COVID-19 schedule and only 1.7% had received a booster dose. By comparison, in the European Economic Area countries during the same period, 72% of people were fully vaccinated against COVID-19 while as many as 51% had received a booster dose [4]. Such a low percentage of vaccinated people in the Ukrainian population may be primarily due to the delayed introduction of vaccines; massive misinformation in the media, probably resulting from the ongoing hybrid war there; online campaigns deprecating vaccines; and incitement by Russian propaganda against health care institutions [3]. Referring to Wadman M. in 2018, the number of measles cases in Europe tripled. Of the 83,000 measles cases across Europe, more than 54,000 were detected in Ukraine. Such a huge increase was the result of political and armed conflicts, delays in the vaccines’ delivery to health institutions, and the growing reluctance to be vaccinated [5]. In Ukraine, the vaccination rate for poliomyelitis with the three-dose vaccine dropped from 91% in 2008 to barely 15% in mid-2015. In 2010, Ukraine was recognized by the WHO as a high-risk country for poliomyelitis. After two unvaccinated children with vaccine-derived polio virus paralysis were identified in August 2015, it was decided to launch three rounds of a nationwide vaccination campaign among children. Ukrainian children are at a higher risk of contracting the polio virus due to lower vaccination rates than in other countries. In 2017, only 48% of children in Ukraine received all three vaccine doses against polio [28]. A nationwide vaccination campaign targeting nearly 140,000 children in Ukraine was scheduled to begin in February 2022 but had to be halted due to the Russian invasion. Because of this situation, some children have not been vaccinated at all or have been vaccinated incompletely [4]. Tuberculosis is prevalent throughout Ukraine; according to a study by Cojocaru et al., the incidence of tuberculosis in the pediatric population is rising [29]. Compared with 2014, there was an 8% increase in new cases among children in 2015. It is estimated that the actual incidence of tuberculosis in Ukraine is 94 cases per 100,000 population. About 25% of cases remain undiagnosed. Multidrug-resistant tuberculosis (MDR-TB) and extensively drug-resistant tuberculosis (XDR-TB) account for an increasing percentage of all tuberculosis cases. Therefore, due to the influx of a large number of refugees and immigrants, TB must not be forgotten, despite the fact that in the WHO European region the rates of this infectious disease are low [29]. In our survey, those whose vaccination decisions regarding their children were influenced by the influx of Ukrainian immigrants were more frequently aware of how the influx of emigrants from Ukraine affected the epidemiological situation in Poland (40/54, 74.07% vs. 346/493, 54.97%; *p* = 0.0021) and were more likely to have been vaccinated due to these circumstances (17/54, 31.48% vs. 9/493, 1.83%; *p* < 0.00001).

Lack of awareness regarding the need for vaccination is one of the main factors contributing to vaccine hesitancy and avoiding mandatory vaccines [30]. Greater knowledge of the disease and its potential consequences is associated with a greater willingness to be vaccinated. This is confirmed by studies regarding influenza [31], meningococcal disease [32], and human papillomavirus [33]. In our survey, respondents whose vaccination decision-making was influenced by the migration of Ukrainians were more likely to believe the vaccination rate with mandatory vaccinations was higher in Poland than in Ukraine (49/54, 90.74% vs. 384/493, 77.89%; *p* = 0. 0014), believed that the incidence rate of diseases covered by mandatory vaccinations was higher in Ukraine than in Poland (48/54, 88.89% vs. 364/493, 73.83%; *p* = 0.0472). This indicates that they were more aware of the current epidemiological situation regarding infectious diseases in both countries.

According to the survey study conducted by Zaprutko T. et al., Poles were significantly more likely to be vaccinated against influenza and COVID-19 than Ukrainians. In addition, Ukrainians were less likely to oppose making these vaccinations mandatory [34]. In our study, participants whose vaccination decisions were influenced by the migration of Ukrainians were more likely to believe the incidence rate of diseases covered by recommended vaccinations was higher in Ukraine than in Poland (49/54, 90.74% vs. 353/493, 71.60%; *p* = 0.0097). There was no significant difference between the two subgroups regarding awareness of the vaccination rate with recommended vaccinations, vaccination rate with SARS-CoV-2 vaccine, and COVID-19 incidence in Poland and Ukraine.

According to Jarret C. et al., dealing with vaccine hesitancy includes increasing vaccination knowledge and awareness [35]. In a study by Lewandowska A. et al., more than half of the parents expressed concern about the supposedly harmful effects of vaccination on their children’s health. Parents reported adverse vaccine reactions (22%), autism (7%), and child’s death (6%) as their main concerns [36]. Negative attitudes towards vaccination are mainly due to lack of knowledge. According to a study by Tang et al., parents are more likely to have their children vaccinated than themselves, especially when the risks of not vaccinating are higher [37]. Referring to Tump et al., people who are confident in their private decisions pay less attention to information coming from the outside [38]. This may explain why the opinion of those who did not express a desire to have their children vaccinated due to the influx of immigrants at the beginning of filling out the questionnaire did not change, while among those who did, the questionnaire was more likely to make them think about having additional vaccinations (41/54, 75. 93% vs. 164/493, 33.27%; *p* < 0.001) and having their children vaccinated with an additional vaccine (44/54, 81.48% vs. 162/493, 32.86%; *p* < 0.0001).

### Limitations

This study has limitations. The questionnaire did not contain restrictions that prevented one person from completing it more than once. The survey was conducted more than six months after the beginning of the influx of migrants from Ukraine. Over time, respondents may have become accustomed to the situation by paying less attention to it. In addition, data were collected through an online survey. The opinions of parents who were very busy and had less access to the internet might have been excluded from this study. However, there are also a few strengths worth mentioning. A large population of people was surveyed and the questions were straightforward and easy to fill (no additional comments from respondents were obtained). In addition, the questionnaire was completely anonymous; therefore, study participants were encouraged and willing to give honest answers.

## 5. Conclusions

The Ukrainian immigrants’ influx affected 9.87% of surveyed parents in their decision to have their children vaccinated. This group was more aware of the differences in infectious diseases’ epidemiologies in Poland and Ukraine. In addition, they had a more positive attitude toward vaccinations and, more often, the survey made them think about getting additional vaccinations for themselves and their children.

## Figures and Tables

**Table 1:** Mandatory and recommended vaccinations in Poland [10].

Mandatory Vaccinations	Recommended Age	Recommended Vaccinations	Recommended Age
BCG—tuberculosis	1 day	Meningococcal disease	After 2 mths
Hepatitis B	1 day, 2 and 7 mths	Varicella	After 13 mths
Rotavirus	2–6 mths	Human Papillomavirus Infection	After 12 yrs
Diphtheria	2, 3–4, 5–6, 16–18 mths, 6, 14, 19 yrs	Tick-borne encephalitis	After 13 mths
Tetanus	2, 3–4, 5–6, 16–18 mths, 6, 14, 19 yrs	Hepatitis A	After 13 mths
Pertussis	2, 3–4, 5–6, 16–18 mths, 6 and 14 yrs	Influenza	After 6 mths or after 2 yrs *
Inactivated Poliovirus Vaccine	3–4, 5–6, 16–18 mths	Coronavirus disease (COVID-19)	After 6 mths
Haemophilus influenzae type B infection	2, 3–4, 5–6, 16–18 mths, 6 yrs		
Pneumococcal disease	2, 4, 13–15 mths		
Measles, Mumps, Rubella	13–15 mths, 6 yrs		

mths—months, yrs—years; * depends on the type of the vaccine.

**Table 2:** Baseline characteristics of parents participating in the study stratified by the influence of Ukrainian immigrants’ influx to Poland in 2022.

Characteristic	Total N = 547	Influenced N = 54	Not Influenced N = 493	*p*-Value
Age in years, median [IQR]	34 [31,39]	33 [29–37.75]	34 [31,39]	0.0293
Female sex, n (%)	503 (91.96)	50 (92.59)	453 (91.89)	0.9309
Place of residence, n (%)				
Rural areas	137 (25.05)	12 (22.22)	125 (25.35)	0.7461
City < 50,000	81 (14.81)	9 (16.67)	72 (14.60)	
City 50,000–100,000	67 (12.25)	9 (16.67)	58 (11.76)	
City 100,000–500,000	83 (15.17)	6 (11.11)	77 (15.62)	
City > 500,000	179 (32.72)	18 (33.33)	161 (32.66)	
Education, n (%)				
Primary	1 (0.18)	0 (0.0)	1 (0.20)	
Vocational	5 (0.91)	1 (1.85)	4 (0.81)	0.7993
Secondary	55 (10.05)	6 (11.11)	49 (9.94)	
Currently studying	14 (2.56)	2 (3.70)	12 (2.43)	
Higher	472 (82.29)	45 (83.33)	427 (86.61)	
Attitude toward vaccination, n (%)				
Positive	497 (90.86)	50 (92.59)	447 (90.67)	0.7489
Negative	7 (1.28)	1 (1.85)	6 (1.22)	
Neutral	43 (7.86)	3 (5.56)	40 (8.11)	
Opinion on usage of recommended vaccination, n (%)				
In favor	482 (88.12)	49 (90.74)	433 (87.83)	0.0428
Against	15 (2.74)	1 (1.85)	14 (2.84)	
No opinion	50 (9.14)	4 (7.41)	46 (9.33)	
Were vaccinated against SARS-CoV-2, n (%)				
4 doses	99 (18.10)	10 (18.52)	89 (18.05)	0.8466
3 doses	266 (48.63)	24 (44.44)	242 (49.09)	
2 doses	115 (21.02)	14 (25.93)	101 (20.49)	
1 dose	15 (2.74)	2 (3.70)	13 (2.64)	
None	52 (9.51)	4 (7.41)	48 (9.74)	
Child vaccinated according to the guidelines, n (%)	537 (98.17)	53 (98.15)	484 (98.17)	0.9891
Child vaccinated with recommended vaccines, n (%)				
Yes	448 (81.90)	47 (87.04)	401 (81.34)	0.0063
No	91 (16.64)	4 (7.41)	87 (17.65)	
Unknown	8 (1.46)	3 (5.56)	5 (1.01)	
Child vaccinated against SARS-CoV-2, n (%)	416 (76.05)	45 (83.33)	371 (75.25)	0.1857
Believed there is a difference in vaccination schedules in Poland and Ukraine, n (%)	507 (92.69)	53 (98.15)	454 (92.09)	0.1045
Impact of migration on vaccination decisions, n (%)				
Already vaccinated	26 (4.75)	17 (31.48)	9 (1.83)	<0.00001
Wanted to get vaccinated	29 (5.30)	15 (27.78)	14 (2.84)	
No impact	492 (89.95)	22 (40.74)	470 (95.33)	
Opinion about statistics concerning vaccination and incidence rate presented in the study, n (%)				
The differences were greater	73 (13.35)	6 (11.11)	67 (13.59)	0.6743
The differences were lesser	141 (25.78)	16 (29.63)	125 (25.35)	
The statistics presented the opposite	11 (2.01)	2 (3.70)	9 (1.83)	
Presented statistics were not surprising	322 (58.87)	30 (55.56)	292 (59.23)	
Awareness of the impact of the influx of immigrants on the epidemiological situation in Poland, n (%)				
Declared awareness	311 (56.86)	40 (74.07)	271 (54.97)	0.0021
Declared knowledge about the situation but no concern	85 (15.54)	10 (18.52)	75 (15.21)	
Lack of awareness	151 (27.61)	4 (7.41)	147 (29.82)	
Our study prompted them to think about getting additional vaccinations, n (%)	205 (37.48)	41 (75.93)	164 (33.27)	<0.0001
Our study prompted them to think about getting additional vaccinations for their children, n (%)	206 (37.66)	44 (81.48)	162 (32.86)	<0.0001

IQR—interquartile range.

**Table 3:** Parent’s knowledge of the current epidemiological situation in Poland and Ukraine.

Vaccination Rate with Mandatory Vaccinations in Poland and Ukraine, n (%)				
Characteristic	Total N = 547	Influenced N = 54	Not Influenced N = 493	*p*-Value
Believed vaccination rate is higher in Poland	433 (79.16)	49 (90.74)	384 (77.89)	0.0014
Believed vaccination rate is higher in Ukraine	14 (2.56)	1 (1.85)	13 (2.64)	
Believed there are no differences	100 (18.28)	4 (7.41)	96 (19.47)	
Level of incidence of diseases that are covered by mandatory vaccinations in Poland and Ukraine, n (%)				
Incidence rate is higher in Poland	13 (2.38)	1 (1.85)	12 (2.43)	0.0472
Incidence rate is higher in Ukraine	412 (75.50)	48 (88.89)	364 (73.83)	
There are no differences	122 (22.12)	5 (9.26)	117 (23.73)	
Vaccination rate with recommended vaccinations in Poland and Ukraine, n (%)				
Vaccination rate is higher in Poland	417 (76.23)	46 (85.19)	371 (75.25)	0.2093
Vaccination rate is higher in Ukraine	19 (3.74)	2 (3.70)	17 (3.45)	
There are no differences	111 (20.29)	6 (11.11)	105 (21.30)	
Level of incidence of diseases covered by recommended vaccinations in Poland and Ukraine, n (%)				
Incidence rate is higher in Poland	17 (3.11)	1 (1.85)	16 (3.25)	0.0097
Incidence rate is higher in Ukraine	402 (73.49)	49 (90.74)	353 (71.60)	
There are no differences	128 (23.40)	4 (7.41)	124 (25.15)	
Vaccination rate with SARS-CoV-2 vaccine in Poland and Ukraine, n (%)				
Vaccination rate is higher in Poland	386 (70.57)	36 (66.67)	350 (70.99)	0.7036
Vaccination rate is higher in Ukraine	24 (4.39)	3 (5.56)	21 (4.26)	
There are no differences	130 (23.77)	15 (27.78)	115 (23.33)	
No answer	7 (1.28)	0	7 (1.42)	
Level of COVID-19 incidence in Poland and Ukraine, n (%)				
Incidence rate is higher in Poland	29 (5.30)	2 (3.70)	27 (5.48)	0.6239
Incidence rate is higher in Ukraine	297 (54.30)	33 (61.11)	264 (53.55)	
There are no differences	211 (38.57)	19 (35.19)	192 (38.95)	
No answer	10 (1.83)	0	10 (2.03)	
Impact of influx of migrants from Ukraine on the incidence rate of the infectious diseases mentioned earlier in Poland, n (%)				
Incidence rate is higher	396 (72.39)	48 (88.89)	348 (70.59)	0.0043
Incidence rate is lower	0	0	0	
There are no differences	151 (27.61)	6 (11.11)	145 (29.41)	

## Data Availability

The data sets used and/or analyzed during the current study can be made available by the corresponding author upon reasonable request.
